# Supplementary material for: Immunogenicity of the 13-Valent Pneumococcal Conjugated Vaccine Followed by the 23-Valent Polysaccharide Vaccine in Chronic Lymphocytic Leukemia
Source: Vaccines (Basel). 2023 Jul 4;11(7):1201. doi: 10.3390/vaccines11071201 (PMC10385862; doi:10.3390/vaccines11071201)
Supplement: Supplementary file 1 [file vaccines-11-01201-s001.zip › vaccines-2468144-supplementary.pdf]

Supplemental data

Table S1. Serologic response in µg/ml in the overall CLL cohort (n=143). Median serotype-specific pneumococcal antibody concentrations prior to and after the sequential PCV13/PPSV23 vaccination schedule are calculated.

	antibody concentration (µg/ml)		
pneumococcal serotypes	prior to vaccination	after vaccination	
	median (IQR)	median (IQR)	p-value
6B	0.06 (0.05-0.25)	0.12 (0.04-0.58)	< 0.001
9V	0.06 (0.04-0.17)	0.23 (0.04-1.10)	< 0.001
14	0.26 (0.06-1.10)	0.83 (0.15-3.20)	< 0.001
19F	0.13 (0.05-0.43)	0.52 (0.10-2.20)	< 0.001
23F	0.09 (0.04-0.36)	0.19 (0.04-1.60)	< 0.001
8	0.12 (0.05-0.46)	0.30 (0.08-1.10)	< 0.001
15B	0.23 (0.07-0.86)	0.41 (0.10-1.60)	< 0.001
20	0.26 (0.09-1.00)	0.41 (0.12-1.70)	< 0.001
33F	0.40 (0.09-1.20)	0.69 (0.15-2.10)	< 0.001
